# Targeted Microchip Capillary Electrophoresis-Orbitrap Mass Spectrometry Metabolomics to Monitor Ovarian Cancer Progression

**DOI:** 10.3390/metabo12060532

**Published:** 2022-06-09

**Authors:** Samyukta Sah, Sylvia R. Yun, David A. Gaul, Andro Botros, Eun Young Park, Olga Kim, Jaeyeon Kim, Facundo M. Fernández

**Affiliations:** 1 School of Chemistry and Biochemistry, Georgia Institute of Technology, Atlanta, GA 30332, USA; ssah9@gatech.edu (S.S.); david.gaul@chemistry.gatech.edu (D.A.G.); 2 Department of Biochemistry and Molecular Biology, Indiana University School of Medicine, Indianapolis, IN 46202, USA; sylyun@iu.edu (S.R.Y.); aabotros@iu.edu (A.B.); epark@truebinding.com (E.Y.P.); olga.kim@nih.gov (O.K.); 3 Indiana University Melvin and Bren Simon Comprehensive Cancer Center, Indianapolis, IN 46202, USA; 4 Petit Institute for Bioengineering and Bioscience, Georgia Institute of Technology, Atlanta, GA 30332, USA

**Keywords:** microchip capillary electrophoresis, mass spectrometry, high-grade serous ovarian cancer

## Abstract

The lack of effective screening strategies for high-grade serous carcinoma (HGSC), a subtype of ovarian cancer (OC) responsible for 70–80% of OC related deaths, emphasizes the need for new diagnostic markers and a better understanding of disease pathogenesis. Capillary electrophoresis (CE) coupled with high-resolution mass spectrometry (HRMS) offers high selectivity and sensitivity for ionic compounds, thereby enhancing biomarker discovery. Recent advances in CE-MS include small, chip-based CE systems coupled with nanoelectrospray ionization (nanoESI) to provide rapid, high-resolution analysis of biological specimens. Here, we describe the development of a targeted microchip (µ) CE-HRMS method, with an acquisition time of only 3 min and sample injection volume of 4nL, to analyze 40 target metabolites in serum samples from a triple-mutant (TKO) mouse model of HGSC. Extracted ion electropherograms showed sharp, baseline resolved peak shapes, even for structural isomers such as leucine and isoleucine. All calibration curves of the analytes maintained good linearity with an average R^2^ of 0.994, while detection limits were in the nM range. Thirty metabolites were detected in mouse serum with recoveries ranging from 78 to 120%, indicating minimal ionization suppression and good accuracy. We applied the µCE-HRMS method to biweekly-collected serum samples from TKO and TKO control mice. A time-resolved analysis revealed characteristic temporal trends for amino acids, nucleosides, and amino acid derivatives. These metabolic alterations are indicative of altered nucleotide biosynthesis and amino acid metabolism in HGSC development and progression. A comparison of the µCE-HRMS dataset with non-targeted ultra-high performance liquid chromatography (UHPLC)–MS results showed identical temporal trends for the five metabolites detected with both platforms, indicating the µCE-HRMS method performed satisfactorily in terms of capturing metabolic reprogramming due to HGSC progression while reducing the total data collection time three-fold.

## 1. Introduction

Ovarian cancer (OC) is the most lethal gynecological malignancy with patients experiencing the highest mortality rate [1]. Due to the asymptomatic tumor growth combined with the lack of effective screening strategies, OC is rarely detected in early stages when the 5-year survival rate is over 90% [2]. Among all OC subtypes, high-grade serous carcinoma (HGSC) is the most common and deadliest subtype, accounting for 70–80% of OC deaths [3]. In approximately 80% of the cases, HGSC is detected at advanced stages, consequently leading to a poor prognosis. Current methods for OC diagnosis include trans-vaginal ultrasound and the measurement of blood CA-125 levels for symptomatic patients [4]. However, these methods lack sensitivity and specificity for early-stage detection [5]. Therefore, new and robust biomarkers are needed to ultimately improve clinical outcomes.

Metabolomics is the analysis of metabolites and other small molecules in biological specimens, providing an instantaneous profile of the molecular phenotype of a biological system under study. Given the limitations of our current knowledge of HGSC progression, metabolomics offers a powerful avenue for understanding disease biology. While liquid and gas chromatography (LC and GC, respectively) and mass spectrometry (MS) remain the major tools in metabolomics studies [6], recent advances in analytical techniques hold promising results to significantly improve metabolite coverage and yield quantitative metabolomics data. One such development is the application of microfluidics to couple capillary electrophoresis (CE) with MS platforms via nanoelectrospray ionization (nanoESI) [7]. CE is particularly well-suited for resolving charged polar metabolites as compounds are separated based on their charge-to-size ratios under a constant electric field [8]. The separation mechanism of CE is fundamentally different from LC, and thus CE-MS offers a complementary analytical tool. Compared to LC, CE provides a higher separation efficiency for ionic metabolites and, when combined with high-resolution mass spectrometry (HRMS), enables the detection of all charged small molecules in the sample [9]. Additionally, CE-MS only requires a few nanoliters of the injection volume [10] and (sub-)microliter sample amounts [11], making it an optimal method for analyzing volume-limited samples that are often excluded from consideration in metabolomics.

Previous studies have demonstrated the use of CE-MS for the metabolomic profiling of plasma [12], urine [13], and tissue samples [14]. For example, a CE-MS metabolomics study profiled over 8000 human plasma samples with an absolute quantification of 94 polar metabolites, showcasing the suitability of CE-MS for large-scale metabolomics [12]. Hirayama et al. applied CE coupled with time-of-flight (TOF) MS for metabolic profiling of colon and stomach cancer tissues [14]. The results from this study demonstrated that CE-MS is a powerful technique for the analysis of primary energy metabolism, revealing cancer-specific metabolic alterations. Significant alterations in the abundance of amino acids, nucleotides, and TCA cycle intermediates were noted as important hallmarks of cancer metabolic reprogramming. A recent study employed a microchip CE (µCE)-HRMS platform to multiplex 35 first- and second-tier biomarkers associated with over 20 newborn screening disorders in a 2-min assay [15].

Here, we analyze 40 key metabolites potentially associated with HGSC progression with a µCE-HRMS method, with an acquisition time under 3 min. The method was validated and applied to serum samples collected biweekly over the entire course of tumor development and progression from triple-mutant (TKO) *p53* ^LSL-R172H/+^ *Dicer1* ^flox/flox^ *Pten* ^flox/flox^ *Amhr2* ^cre/+^ animals [16], which is a mouse model that reproduces the clinical progression of human HGSC with close phenotypic, histological, and molecular similarities. The results from this study showcase the use of µCE-HRMS as a high-throughput platform to capture granular time-course information from ovarian cancer animal models, thereby providing new opportunities to understand the molecular course of the disease.

## 2. Results

### 2.1. µCE-HRMS Method in Full Scan Mode

**Figure 1:**
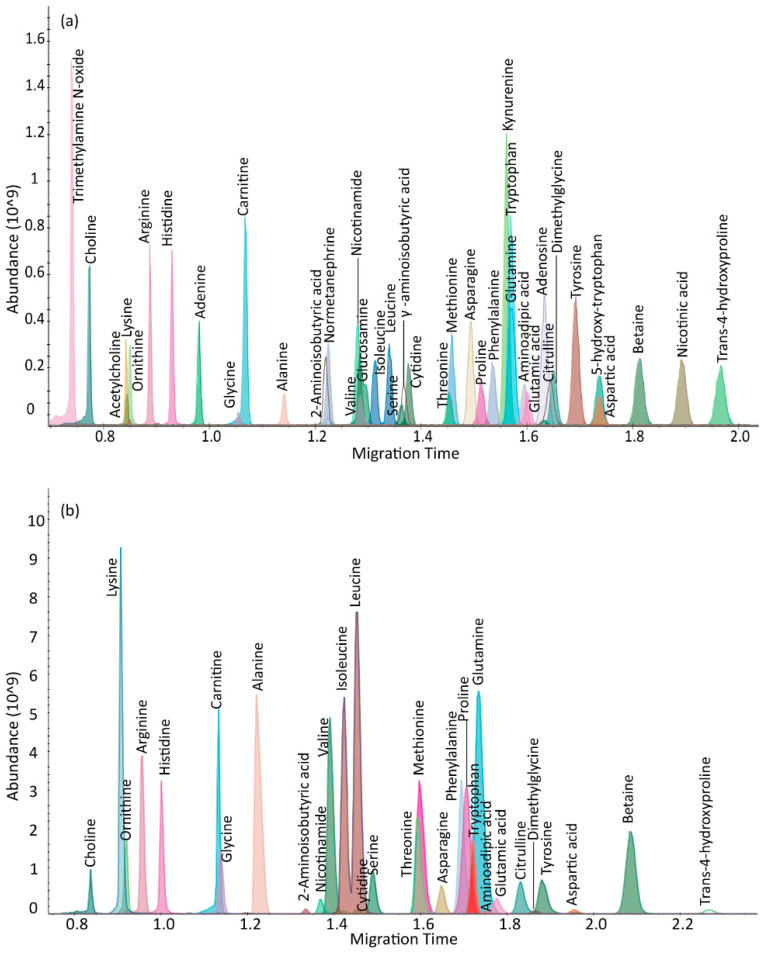
µCE-HRMS analysis in full scan mode. Extracted ion electropherogram (XIE) for (**a**) 40 target metabolites analyzed in a synthetic mixture and (**b**) 30 metabolites detected in a TKO mouse serum extract sample. The *x*-axis represents the migration time in minutes and the *y*-axis shows the MS peak abundance.

µCE-HRMS measurements used the full scan mode in the Q Exactive plus mass spectrometer to analyze 40 metabolites in a standard mixture with a 3-min acquisition time. The metabolites targeted in this study were selected from a list of biomarkers that are potentially important for following cancer progression [17]. These included amino acids, amino acid derivatives, vitamins, alkylamines, and nucleotides (Table 1 and Table S1). The migration times for the target metabolites ranged from 0.8 to 2.0 min; trimethylamine-N-oxide, a basic charged metabolite, was the first compound to migrate through the chip channel, and trans-4-hydroxyproline was the last. The peak widths of the analytes ranged between 1–3 s, which was sufficient to collect more than 10 data points across each peak in full scan mode at the highest scan speed and lowest resolving power setting (12 Hz and 17,500, respectively) of the Orbitrap mass spectrometer. The experiments were conducted at a resolving power setting of 35,000, which reduced the number of MS data points by almost half for a 2.4 s wide CE peak (Figure S1), thereby suggesting that the optimum CE peak shapes for accurate quantification were best achieved at a 17,500 setting. A typical extracted ion electropherogram (XIE) for a mixture of 40 metabolites showed sharp, Gaussian-like peak shapes for most analytes (Figure 1a). In addition, structural isomers such as leucine and isoleucine were baseline resolved.

Linearity statistics, migration time analysis, and detection limits for the target metabolites were calculated and presented in Table 1. The detection limits ranged from 3 to155 nM; for 80% of the analytes, the detection limits were below 60 nM. Compounds with quaternary amine groups such as carnitine and choline had lower detection limits of 3 nM and 7 nM, respectively, showcasing the high sensitivity achieved by µCE-HRMS for charged polar compounds. The data acquired were highly linear in the tested concentration range. The tested range was optimized according to the analyte concentration in the serum samples. Most analytes had R^2^ value > 0.990 (R^2^ 0.994 on average). The lowest R^2^ value was 0.982 for asparagine, which was likely due to the differences in the ionization efficiency between asparagine and ^13^C_6_ arginine—which was used as the internal standard. On the same note, arginine, methionine, and phenylalanine had R^2^ values of 0.999, 0.999, and 0.997, respectively, due to minimal differences in the ionization efficiency between the analytes and the corresponding internal standards (^13^C_6_ arginine, ^13^C methionine D_3_, and ^13^C phenylalanine). The RSD for analyte migration times was 2% (*n* = 18) on average, indicating an excellent robustness of the analyte separation in the µCE-HRMS method. Choline and trimethylamine-*n*-oxide experienced some tailing and had a slightly higher % RSD of 3 and 3.4%, respectively.

### 2.2. µCE-HRMS Method Validation in TKO Mouse Serum Samples

The µCE-HRMS method was validated in serum samples from a TKO mouse model of HGSC. Thirty metabolites were detected in TKO serum samples (Figure 1b, Table 2) and their identities were confirmed by spiking standard compounds in the extracts. The percent recoveries of the standard compounds from the serum matrix ranged from 78–120% (Table 2). The ion suppression or ion enhancement effects observed for some analytes was acceptable and likely due to co-eluting compounds in the serum matrix [18]. Choline and ornithine had the lowest % recovery of 78%, whereas for some metabolites, including alanine, dimethylglycine, and hydroxyproline, the % recoveries were close to 100%. In general, acceptable % recoveries were achieved for most detected compounds, indicating minimal ionization suppression effects by µCE-HRMS. The migration time statistics and peak area reproducibility for metabolites detected in TKO mouse serum were also evaluated. Data acquired for a TKO serum sample indicated good reproducibility for all measured metabolites. The % RSD for peak areas for *n* = 23 runs were between 1–15% (Table 2). Only choline had a % RSD of 19%, which was likely due to peak tailing. Furthermore, the migration time statistics for TKO serum samples were calculated. For this particular dataset, a shift in the migration time was observed for 5 µCE-HRMS runs collected immediately after a BGE refresh (Figure S2). Therefore, to correct for this variation, the ratio between the migration time of the target analyte and their respective internal standard was used for analysis. The RSD for the migration time for *n* = 23 injections ranged from 0.10 to 2.66% (Table 2). Overall, good reproducibility, sensitivity, and linearity were attained by µCE-HRMS for targeted metabolomics purposes.

### 2.3. Application to Sequentially Collected TKO Mice Serum Samples

**Figure 2:**
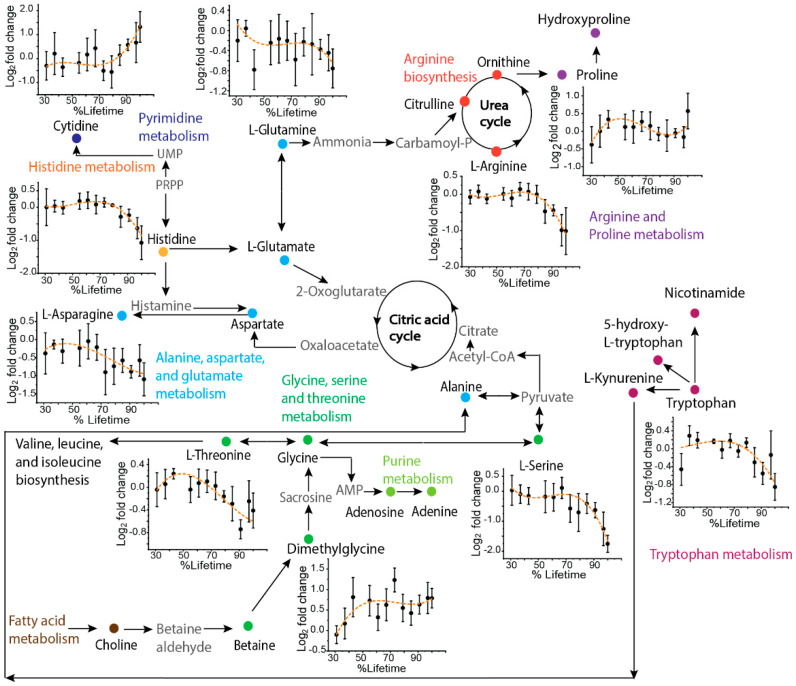
Pathway map showing a selection of key metabolites analyzed by µCE-HRMS that track HGSC progression. Metabolites targeted in this study are shown as solid symbols and intermediates connecting the pathways are shown in grey text. Time-resolved serum metabolic trajectories for TKO mice are shown for select metabolites. For time-resolved plots, the *x*-axis shows the % lifetime, calculated as the ratio of the age of the mouse at a given serum sampling time point compared to the total lifespan of that specific animal. The *y*-axis shows the fold change calculated as the base 2 logarithm of the abundance in TKO/TKO control samples. Positive values indicate higher serum levels in TKO animals and negative values indicate lower serum levels in TKO animals compared to TKO controls. Error bars represent the standard error of the log_2_ fold change between TKO and control samples.

The µCE-HRMS method was applied to collect granular time-course metabolic information from the TKO mouse model of HGSC. Sequentially collected serum samples from three TKO (*n* = 33) and three TKO control mice (*n* = 39) were analyzed, and the time-resolved changes for the targeted metabolites were investigated to gain insight into the altered pathways associated with HGSC progression. A schematic showing the metabolic pathways that were targeted, along with the trajectories for metabolites that showed characteristic temporal trends in TKO mice, is presented in Figure 2. The time courses for amino acids such as arginine, histidine, threonine, serine, tryptophan, and asparagine showed a decreasing temporal trend, while cytidine and dimethylglycine showed an increasing abundance over time for TKO mice compared to TKO controls. Altered abundances of these metabolites have been reported in other cancer studies [17], including ovarian cancer [19,20]. A decreased level of histidine is among one of the most frequently reported amino acid alterations observed in OC [21]. Histidine is likely related to nucleotide biosynthesis via 5-phosphoribosyl-1-pyrophosphate (PRPP) [22], a crucial substrate for histidine, tryptophan, purine, and pyrimidine biosynthesis. Thus, the decreasing temporal trend for histidine and the increasing abundance of cytidine observed in TKO mice could be indicative of the disturbed PRPP-medicated histidine-nucleotide pathway in HGSC. Additionally, the amino acid glutamine is also involved in nucleotide metabolism [23], and reduced levels of glutamine have frequently been reported in previous OC studies [19,24]. The decreasing temporal trend observed for glutamine in our study is in line with the aforementioned studies, which further emphasizes the potential importance of nucleotide biosynthesis in HGSC development. Additionally, glutamine metabolism is linked to arginine biosynthesis, the arginine-citrulline-nitric oxide cycle, and the urea cycle (Figure 2) [19]. A decreasing temporal trend for arginine was observed in TKO mice. Arginine is known to play a well-established role in tumor proliferation and metastasis [25], and therefore the observed alterations here indicate a potentially important role of arginine in HGSC progression. Furthermore, changes in serine, threonine, and dimethylglycine levels suggest that the glycine, serine, and threonine pathway, which is also the source of one-carbon metabolism [26], is likely an important pathway of interest for HGSC. Although the observed temporal trends need to be validated with a larger sample size, the results here serve as a useful indicator of µCE-HRMS being able to capture the metabolic reprogramming associated with HGSC progression.

**Figure 3:**
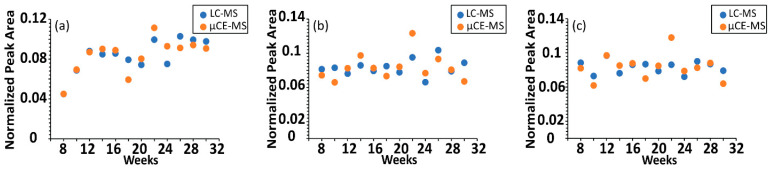
Comparison of time-resolved serum metabolomics data acquired with UHPLC-MS and µCE-HRMS platforms. Normalized peak abundance of (**a**) serine, (**b**) threonine, and (**c**) citrulline in a TKO control mouse. UHPLC-MS abundances are shown in blue and µCE-HRMS data are shown in orange. For visualization purposes, data were normalized to the sum of the peak areas for all time points.

Additionally, the time-course trajectories of serine, threonine, citrulline, ornithine and tryptophan—5 out of the 40 metabolites targeted in this study—have been previously characterized by non-targeted ultra-high performance liquid chromatography (UHPLC)-MS [27]. A comparison of the µCE-HRMS dataset with previously published time-resolved UHPLC-MS data revealed that µCE-HRMS was able to capture identical temporal trends for all five analytes (Figure 3 and Figure S3)—only the µCE-HRMS data for ornithine was slightly noisier than the UHPLC-MS data. This was likely due to the ion suppression effect noted earlier for ornithine (Table 2). Nevertheless, time-resolved data collected by the µCE-HRMS platform produced informative time-course traces while reducing the total analysis time three-fold. The higher sample throughput should prove beneficial when investigating very large animal or human cohorts.

### 2.4. µCE-HRMS Coupled with Parallel Reaction Monitoring MS

**Figure 4:**
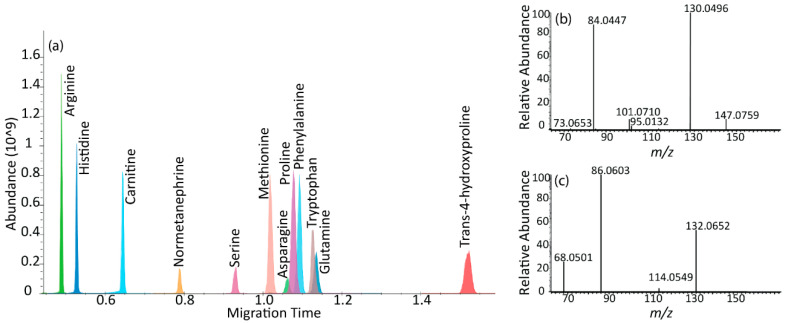
µCE-MS/MS separation. (**a**) Extracted ion electropherogram (XIE) of 12 metabolites in a scheduled and multiplexed PRM method. The *x*-axis represents the migration time in minutes and the *y*-axis shows the MS peak abundance. (**b**) PRM transitions for glutamine with precursor *m/z* 147.0759 at HCD 35 (**c**) PRM transitions for trans-4-hydroxyproline with precursor *m/z* 132.0652 at HCD 35. Most important, MS method parameters: AGC target value 1E5 and maximum ion injection time of 20 ms. Spectral multiplexing (MSX) count of 6.

µCE-HRMS experiments were also conducted in the parallel reaction monitoring (PRM) mode to assess the use of PRM MS coupled to µCE separations for large-scale targeted metabolomics studies. In general, the PRM mode can offer higher selectivity and sensitivity than full scan mode [28] by monitoring all fragments from a selected precursor ion and by enhancing the signal-to-noise ratios. Preliminary experiments in the PRM mode showed that the Orbitrap mass analyzer scan speed was not sufficient to collect enough datapoints across the narrow µCE peaks (1–2 s) and produced only 3–5 MS scans per peak (Figure S4). Alternatively, a multiplexed and scheduled PRM method was developed to reduce the MS cycle time [29], which successfully acquired 7–15 data points across 1–3-s-wide peaks for 12 analytes under 3 min (Table S2). The XIE for the 12 analytes and corresponding MS/MS spectra for glutamine and trans-4-hydroxyproline are shown in Figure 4. Good reproducibility was observed for this method: the % RSD for peak areas was 7–14% (*n* = 4) without the use of internal standards (Table S2). However, the developed PRM strategy could only be applied to a maximum of 12 analytes without increasing the total analysis time (Figure S5), which limited the throughput of the assay. Therefore, our results suggest that full scan Orbitrap MS is the optimal scan mode for targeted metabolomics studies with many targets using this µCE-HRMS platform.

## 3. Discussion

Over the past decade, MS-based metabolomics methods have become one of the primary tools for examining cancer-related metabolic alterations and identifying potential biomarkers. In this study, we investigated the suitability of µCE-HRMS for high-throughput targeted metabolomics profiling of longitudinally collected serum samples from the TKO *p53 Dicer1* mouse model of HGSC. This system is an integrated CE and nanoESI platform, thereby providing rapid, sensitive, and high-resolution analyses of biological samples. Forty metabolites from key metabolic pathways associated with cancer progression were analyzed with a 3-min acquisition time method, allowing for a comprehensive examination of multiple biosynthetic pathways. This method provided the precise quantification and high-resolution separations for positively charged polar compounds. Besides, the targeted µCE-HRMS method yielded data comparable to non-targeted UHPLC-MS collected in our previous study.

However, while this method offers many advantages, there are also some limitations. Although many metabolites were analyzed in a short period of time, CE-MS separations in this instrument can only be conducted in the positive ionization mode at this time, and therefore some metabolite classes including organic acids, fatty acids, alcohols, and sugars were not detectable. Furthermore, though the total acquisition time is 3-min per run, there is an additional 1–2 min needed for rinsing the sample in between samples. This, however, only applies to the ZipChip system without the autosampler. Also, a BGE refresh is recommended after every 5 injections, which requires 1–2 min to complete. Another limitation is that this method could only be performed in full scan mode at a 17,500 resolving power and required the use of internal standards to achieve an acceptable reproducibility for the targeted metabolites (%RSD < 20%). The %RSD of peak areas without internal standard normalization was 15–28% (Table 2). Although three isotopically labeled internal standards were used to correct for the ionization suppression or enhancement for all analytes, the method accuracy and precision can be further improved by incorporating the corresponding internal standards for all metabolites (the %RSD for arginine/^13^C_6_ arginine was 2.85%, phenylalanine/^13^C phenylalanine 1.45%, and methionine/^13^C methionine D_3_ was 1.11%).

## 4. Materials and Methods

### 4.1. Chemicals

LC-MS grade methanol and water were purchased from Fisher Chemical (Fisher Scientific International, Inc. Pittsburgh, PA, USA). The ammonium acetate used for preparing the sample diluent was purchased from Sigma-Aldrich (Saint Louis, MO, USA). All measured standard compounds (Table 1 and Table S1) were purchased from Sigma-Aldrich (Saint Louis, MO, USA). Isotopically labeled ^13^C_6_ arginine, ^13^C methionine D_3_, and ^13^C phenylalanine were purchased from Cambridge Isotope Laboratories (Tewksbury, MA, USA) and were used as internal standards. The metabolites background electrolytes (BGE) kit was purchased from 908 devices (Boston, MA, USA) and prepared according to the manufacturer protocol.

### 4.2. Triple Mutant (TKO) Mouse and Serum Sampling

Serum samples were collected from triple-mutant (TKO) p53-Dicer1-Pten mice as described elsewhere [16,27]. Briefly, TKO p53^LSL-R172H/+^ *Dicer1*^flox/flox^ *Pten*^flox/flox^ *Amhr2*^cre/+^ mice were generated by mating *p53*^LSL-R172H/+^*Dicer1*^flox/flox^*Pten*^flox/flox^ female mice with *Dicer1*^flox/flox^*Pten*^flox/flox^*Amhr2*^cre/+^ male mice. Female *p53*^LSL-R172H/+^*Dicer1*^flox/flox^*Pten*^flox/flox^ mice were used as TKO controls. TKO control mice carry the same genetic makeup as TKO mice but do not develop HGSC. Biweekly serum collection from TKO and TKO control mice occurred starting at 8 weeks of age until a humane end point for sacrifice or development of ascites. TKO mice were sacrificed at Indiana University School of Medicine in accordance with lab animal protocols (21124) approved by the IACUC.

### 4.3. Sample Preparation

#### 4.3.1. Calibration Samples

To improve CE peak shape and support electrophoretic focusing, a sample diluent consisting of 133 mM ammonium acetate and 0.1% formic acid was prepared [30]. This sample diluent was spiked with 1 µM ^13^C phenylalanine, 3 µM ^13^C_6_ arginine, and 0.8 µM ^13^C methionine D_3_ as internal standards. One mM solutions of each target analyte were prepared in 1:1 methanol/water and were used for prepping stock standard mixture solutions. Calibration mixtures were prepared from serial dilutions of stock mixture solutions (6 to 8 serial dilutions) using the spiked sample diluent in a 1:4 ratio. Each calibration standard was analyzed twice to yield calibration curves, calculate figures of merit, and perform metabolite quantification. Quantitation was performed with the analyte peak areas relative to the peak area of one of the three isotopically labeled internal standards (^13^C_6_ arginine, ^13^C methionine D_3_, and ^13^C phenylalanine) chosen based on migration time similarities and the reproducibility of peak areas. Further details for the metabolite standards and internal standards used are given in Tables S1 and S3.

#### 4.3.2. Serum Sample Preparation

Polar metabolites were extracted from serum samples as described in our previous study. Briefly, an extraction solvent consisting of a mixture of isotopically labeled internal standards including 808 µM ^13^C_6_ arginine and 212 µM ^13^C methionine D_3_ was added to methanol in a 1:60 ratio and stored at 4 °C until further use. Serum samples were thawed on ice, followed by the addition of extraction solvent in a 1:3 ratio to 10 µL serum aliquot to precipitate proteins. The samples were vortexed for 30 s and centrifuged at 13,000 rpm for 7 min. To enhance CE peak shape, the resulting supernatant was diluted in a 1:4 ratio with the sample diluent containing 133 mM ammonium acetate, 0.1% formic acid, and 1 µM ^13^C phenylalanine. A quality control (QC) sample was prepared by pooling 2 µL aliquots of the serum extracts and was used to monitor the instrument stability through the course of the experiment.

### 4.4. µCE-HRMS Analysis

µCE-HRMS analyses were performed using the microchip ZipChip^®^ capillary electrophoresis system (classic interface, 908 Devices, Boston, MA, USA), which was coupled to a high-resolution accurate mass Q Exactive plus mass spectrometer (Thermo Fisher Scientific, Waltham, MA, USA). µCE-HRMS separations used ZipChip^®^ HS chips (10 cm channel). Preliminary experiments surveyed the effect of different custom background electrolytes (BGE), such as 60/40 water/isopropanol with 1% formic acid and 50/50 acetonitrile/water with 0.75% formic acid, and the metabolites BGE Kit (908 devices, Boston, MA, USA) on peak shape and electrospray stability. BGE was prepared using the metabolites BGE Kit (908 Devices), which provided the most stable electrospray and was therefore used for all experiments. The BGE was composed of 68% water, 20% isopropanol, 10% acetonitrile, and 2% formic acid with a pH of 2.2. For CE separations, a field strength of 1000 V/cm was applied, and the injection volume was set to 4 nL. The µCE system when operated without an autosampler requires a minimum sample volume of 10 µL to be loaded in the chip sample well. The total analysis time was 3 min and the pressure assist feature was activated at 2.5 min to clear out any slow migrating compounds present in serum samples. Q Exactive plus data were acquired in full scan mode, except when noted otherwise. All experiments were performed in the positive ionization mode in the 50–500 *m/z* range at a mass resolution setting of 17,500. The capillary temperature was set to 200 °C and the sheath gas flow rate was 2 psi. The automatic gain control (AGC) target value was set to 3E6 and the maximum injection time was 20 ms. The metabolite identities were confirmed by accurate mass measurements and by spiking samples with the target metabolites.

µCE-HRMS/MS experiments were performed with a scheduled and multiplexed parallel reaction monitoring (PRM) method. The AGC target value was 1E5 and the maximum ion injection time was 20 ms. The spectral multiplexing count (MSX) was set to 6, and a normalized collision energy of 35 was applied to fragment the precursor ions in the HCD cell, followed by an Orbitrap analysis at a 17,500-mass resolution setting.

Data were acquired using Xcalibur 3.0 (Thermo Scientific) and were imported to Skyline software [31] for peak picking and integration. The peak picking procedure used the analyte accurate *m/z* and migration time. Peak areas obtained from Skyline were exported as spreadsheets for further analysis. Peak areas for the isotopically labeled ^13^C phenylalanine were manually corrected by subtracting the second isotope abundance of the unlabeled phenylalanine.

### 4.5. Method Validation

The full scan mode µCE-HRMS method was validated by linearity, limit of detection (LOD), limit of quantification (LOQ), recovery of metabolite standards in the serum matrix, and method precision for each targeted metabolite detected in the TKO serum samples. LOD and LOQ were calculated as three-times the standard deviation of the calibration curve y-intercept divided by its slope (3 × SD_yintercept_/slope), and 10 × SD_yintercept_/slope, respectively. The method precision for all analytes was evaluated by monitoring the relative standard deviation (RSD) of peak areas and migration time for the detected metabolites in TKO serum across *n* = 23 injections.

### 4.6. Time-Resolved TKO Serum Metabolomics Experiments

The µCE-HRMS method was applied to sequentially collect serum samples from three TKO (*n* = 33) and three TKO control (*n* = 39) mice (Table S4). The temporal trends of the targeted metabolites at premalignant stages, tumor initiation stages, early stages, and advanced stages until mouse death were assessed to identify trends associated with HGSC progression. Data were aligned using a percent (%) lifetime variable. Briefly, due to the individual differences between TKO animals, the variability in the time-course of HGSC was observed in our dataset. Therefore, a variable termed % lifetime, which is the normalized time until death for each mouse, was calculated as follows:

$$\%\mathrm{Lifetime}\;=\;\frac{\mathrm{Age}\;\mathrm{of}\;\mathrm{mouse}}{\mathrm{Total}\;\mathrm{lifespan}\;\mathrm{or}\;\mathrm{the}\;\mathrm{age}\;\mathrm{at}\;\mathrm{the}\;\mathrm{last}\;\mathrm{time}\;\mathrm{point}\;\mathrm{of}\;\mathrm{blood}\;\mathrm{collection}\;}\times 100\%$$

## 5. Conclusions

In conclusion, we evaluated the analytical performance of a targeted µCE-HRMS method and demonstrated the successful profiling of serum metabolic alterations in murine models of HGSC. Overall, the µCE-HRMS method provided acceptable reproducibility and sensitivity for target analytes, while minimizing sample consumption, solvent use, and total analysis time. An analysis of longitudinally collected serum samples resulted in characteristic temporal trends for amino acids, amino acid derivatives, and nucleosides. These metabolic alterations are indicative of disturbed nucleotide biosynthesis and amino acid metabolism in HGSC. Although there are some limitations with this method, it can be employed as a complementary technique to traditional LC-MS methods to further improve metabolite coverage. Future experiments to investigate the suitability of µCE-MS/MS to improve the assay selectivity should be conducted by coupling the CE system with faster scanning instruments such as time-of-flight MS.

## Figures and Tables

**Table 1:** Linearity, detection limits, limits of quantification, and migration time analysis for target metabolites in a standard mixture. Isotopically labeled ^13^C_6_ arginine, ^13^C methionine D_3_, and ^13^C phenylalanine were spiked as internal standards. Detection limits and limits of quantification were calculated as three times the standard deviation of the y intercept divided by the slope (3 × SD_yintercept_/slope) and 10 × SD_yintercept_/slope, respectively.

Metabolite Name	Migration Time (min)	Migration Time % RSD (*n* = 18)	Detection Limit (nM)	Absolute limit of Detection (moles)	Limit of Quantification (nM)	R^2^	Tested Concentration Range (µM)	Metabolite Classification
5′-Hydroxy-L-tryptophan	1.8	1.7	58.4	2.3 × 10^−16^	194.8	0.993	0.25–5	Amino acid
Acetylcholine	0.9	2.8	17.8	7.1 × 10^−17^	59.5	0.990	0.066–2	Neurotransmitters
Alanine	1.2	2.0	19.1	7.6 × 10^−17^	63.6	0.997	0.05–25	Amino Acids
2-Aminoisobutyric acid	1.3	2.0	64.8	2.6 × 10^−16^	216.0	0.994	0.25–5	Amino Acids
Arginine	0.9	2.5	51.4	2.1 × 10^−16^	171.4	0.999	0.1–25	Amino Acids
Asparagine	1.5	1.8	34.8	1.4 × 10^−16^	116.1	0.982	0.1–25	Amino Acids
Betaine	1.9	1.6	11.5	4.6 × 10^−17^	38.2	0.999	0.033–10	Amino Acids
Carnitine	1.1	2.2	2.5	1.0 × 10^−17^	8.5	0.989	0.02–3	Alkylamines
Choline	0.8	3.0	6.8	2.7 × 10^−17^	22.5	0.999	0.05–25	Vitamins
*n*,*n*-Dimethylglycine	1.7	1.7	51.0	2.0 × 10^−16^	170.0	0.998	0.1–2	Vitamins
Glucosamine	1.3	2.1	29.6	1.2 × 10^−16^	98.7	0.994	0.16–5	Amino Sugar
Glutamine	1.6	1.8	69.3	2.8 × 10^−16^	231.1	0.997	0.25–25	Amino Acids
Glycine	1.1	2.0	27.0	1.1 × 10^−16^	89.9	0.998	0.25–50	Amino Acids
Histidine	1.0	2.4	19.5	7.8 × 10^−17^	65.1	0.996	0.1–25	Amino Acids
Trans-4-hydroxy-L-proline	2.0	1.3	55.5	2.2 × 10^−16^	185.0	0.991	0.1–2	Amino Acids
Methionin × 10	1.5	1.9	23.3	9.3 × 10^−17^	77.8	0.999	0.1–25	Amino Acids
Normetanephrine	1.3	2.2	16.8	6.7 × 10^−17^	55.9	0.989	0.05–1	Neurotransmitters
Phenylalanine	1.6	1.9	17.4	7.0 × 10^−17^	58.1	0.997	0.25–25	Amino Acids
Proline	1.6	1.8	24.4	9.8 × 10^−17^	81.4	0.996	0.25–25	Amino Acids
Serine	1.4	1.9	31.0	1.2 × 10^−16^	103.3	0.991	0.1–25	Amino Acids
Threonine	1.5	1.8	45.0	1.8 × 10^−16^	150.1	0.999	0.1–25	Amino Acids
Trimethylamine-*n*-oxide	0.8	3.4	83.5	3.3 × 10^−16^	278.2	0.989	0.25–10	Organic Oxoazanium Compounds
Tryptophan	1.6	1.9	40.6	1.6 × 10^−16^	135.3	0.993	0.1–25	Amino Acids
Tyrosine	1.7	1.8	80.0	3.2 × 10^−16^	266.8	0.988	0.25–25	Amino Acids
Valine	1.3	2.0	7.5	3.0 × 10^−17^	24.9	0.997	0.05–25	Amino Acids
Nicotinamide	1.3	1.7	17.7	7.1 × 10^−17^	59.1	0.994	0.1–25	Vitamins
Aspartic acid	1.8	1.7	21.0	8.4 × 10^−17^	70.0	0.992	0.1–10	Amino Acids
Nicotinic acid	1.9	1.5	22.3	8.9 × 10^−17^	74.2	0.996	0.05–1	Vitamins
γ-Aminobutyric acid	1.0	2.3	65.2	2.6 × 10^−16^	217.3	0.994	0.25–5	Amino Acids/Neurotransmitters
Aminoadipic acid	1.6	1.8	30.3	1.2 × 10^−16^	101.0	0.998	0.1–2	Amino acids
Cytidine	1.4	2.0	13.2	5.3 × 10^−17^	44.0	0.998	0.05–25	Pyrimidines
Citrulline	1.7	1.8	29.3	1.2 × 10^−16^	97.8	0.994	0.1–25	Amino Acids
Kynurenine	1.6	1.8	154.8	6.2 × 10^−16^	516.0	0.998	0.5–5	Amino acid
Isoleucine	1.3	2.0	14.3	5.7 × 10^−17^	47.7	0.997	0.05–25	Amino Acids
Leucine	1.4	2.0	8.7	3.5 × 10^−17^	29.0	0.997	0.05–25	Amino Acids
Ornithine	0.9	2.6	17.9	7.2 × 10^−17^	59.5	0.998	0.05–25	Amino Acid
Lysine	0.9	2.0	10.6	4.2 × 10^−17^	35.5	0.994	0.25–25	Amino Acids
Glutamic acid	1.6	1.8	39.9	1.6 × 10^−16^	132.8	0.998	0.1–10	Amino Acids
Adenosine	1.7	1.6	96.3	3.9 × 10^−16^	321.0	0.984	0.25–5	Purines
Adenine	1.0	2.2	9.7	3.9 × 10^−17^	32.3	0.991	0.03–0.6	Purines

**Table 2:** µCE-HRMS targeted metabolomics assay method validation in TKO mouse serum samples. Percent recovery was calculated using peak areas as follows: (peak area from spiked TKO serum–peak area of TKO serum)/peak area from neat standard mixture × 100%. RSD of peak areas with and without the normalization against the peak areas of the internal standards are given.

Metabolite Name	Experimental *m/z*	Mass Error (ppm)	Migration Time (min)	%RSD Migration Time (*n* = 23)	Percent Recovery	%RSD for Peak Area without Internal Standard Correction (*n* = 23)	%RSD for Peak Area Corrected with Internal Standard (*n* = 23)
Alanine	90.0553	2.22	1.2	0.94	98	17.24	6.07
2-Aminoisobutyric acid	104.0709	2.88	1.3	0.84	108	21.92	5.02
Arginine	175.1190	0.57	1.0	0.13	109	20.86	2.85
Asparagine	133.0608	0.00	1.6	2.35	98	25.92	13.37
Betaine	118.0864	0.85	1.9	0.57	90	17.14	6.88
Carnitine	162.1123	−0.62	1.2	1.32	96	15.12	11.59
Choline	104.1073	2.88	0.9	1.23	78	27.72	19.39
Dimethylglycine	104.0709	2.88	1.8	0.52	100	18.22	4.83
Glutamine	147.0762	−1.36	1.7	2.53	93	20.47	10.95
Glycine	76.0397	5.26	1.2	1.66	120	20.38	8.15
Histidine	156.0767	−0.64	1.1	0.38	119	22.35	9.59
Trans-4-hydroxyproline	132.0655	0.00	2.0	0.62	102	21.22	8.47
Methionine	150.0582	−0.67	1.5	0.10	116	20.61	1.11
Phenylalanine	166.0862	−0.60	1.6	0.10	107	20.65	1.45
Proline	116.0707	0.86	1.6	0.54	116	17.94	4.55
Serine	106.0501	1.89	1.5	2.06	120	27.30	13.09
Threonine	120.0657	1.67	1.5	2.32	117	23.20	8.34
Tryptophan	205.0971	−0.49	1.6	0.31	94	24.01	7.27
Tyrosine	182.0812	0.00	1.8	0.49	110	25.05	10.09
Valine	118.0864	1.69	1.4	0.44	102	20.60	4.66
Nicotinamide	123.0556	2.44	1.5	0.70	105	21.28	4.48
Aspartic acid	134.0448	0.00	1.8	2.66	118	26.43	12.96
Aminoadipic acid	162.0759	−1.23	1.7	0.28	97	21.31	8.25
Cytidine	244.0927	−0.41	1.5	2.57	107	27.08	14.47
Ornithine	133.0971	−0.75	1.0	0.35	78	24.58	12.45
Citrulline	176.1030	0.57	1.7	0.42	93	24.19	11.13
Isoleucine	132.1018	−0.76	1.4	0.40	112	23.18	6.99
Leucine	132.1018	−0.76	1.4	0.52	106	22.26	6.21
Lysine	147.1126	−1.36	1.0	0.31	119	22.24	13.74
Glutamic acid	148.0603	−0.68	1.7	0.26	115	23.87	11.74

## Data Availability

Data generated in this study are available through the NIH Metabolomics Workbench (http://www.metabolomicsworkbench.org/) with project ID PR001353 (DOI: http://dx.doi.org/10.21228/M8TQ43).

## Supplementary Materials

The following supporting information can be downloaded at: https://www.mdpi.com/article/10.3390/metabo12060532/s1, Figure S1. Comparison of µCE-HRMS peak shapes at different Orbitrap mass resolving power settings. Figure S2. Analysis of migration time variation for metabolite analytes in TKO mouse serum. Migration time for ^13^C phenylalanine across 23 replicates. Figure S3. Comparison of time-resolved serum metabolomics data acquired with UHPLC-MS or µCE-HRMS platforms. Figure S4. Comparison of µCE-HRMS peak shapes in different MS scan modes. Figure S5. µCE-MS/MS separation of metabolites of interest. Table S1: Metabolite standards and internal standards used for µCE-MS/MS analyses. Table S2: Multiplexed/scheduled µCE-MS/MS method. Table S3. Migration times and relative standard deviation (RSD) for peak areas of target analytes in TKO serum. Table S4. TKO mouse cohort information.
