# Supplementary material for: Targeted Microchip Capillary Electrophoresis-Orbitrap Mass Spectrometry Metabolomics to Monitor Ovarian Cancer Progression
Source: Metabolites. 2022 Jun 9;12(6):532. doi: 10.3390/metabo12060532 (PMC9230880; doi:10.3390/metabo12060532)
Supplement: Supplementary file 1 [file metabolites-12-00532-s001.zip › metabolites-1748079-supplementary.pdf]

Supplementary Materials for

Targeted Microchip Capillary Electrophoresis-Orbitrap Mass Spectrometry Metabolomics to Monitor Ovarian Cancer Progression.

Samyukta Sah, Sylvia R. Yun, David A. Gaul, Andro Botros , Eun Young Park, Olga Kim, Jaeyeon Kim, Facundo M. Fernández.

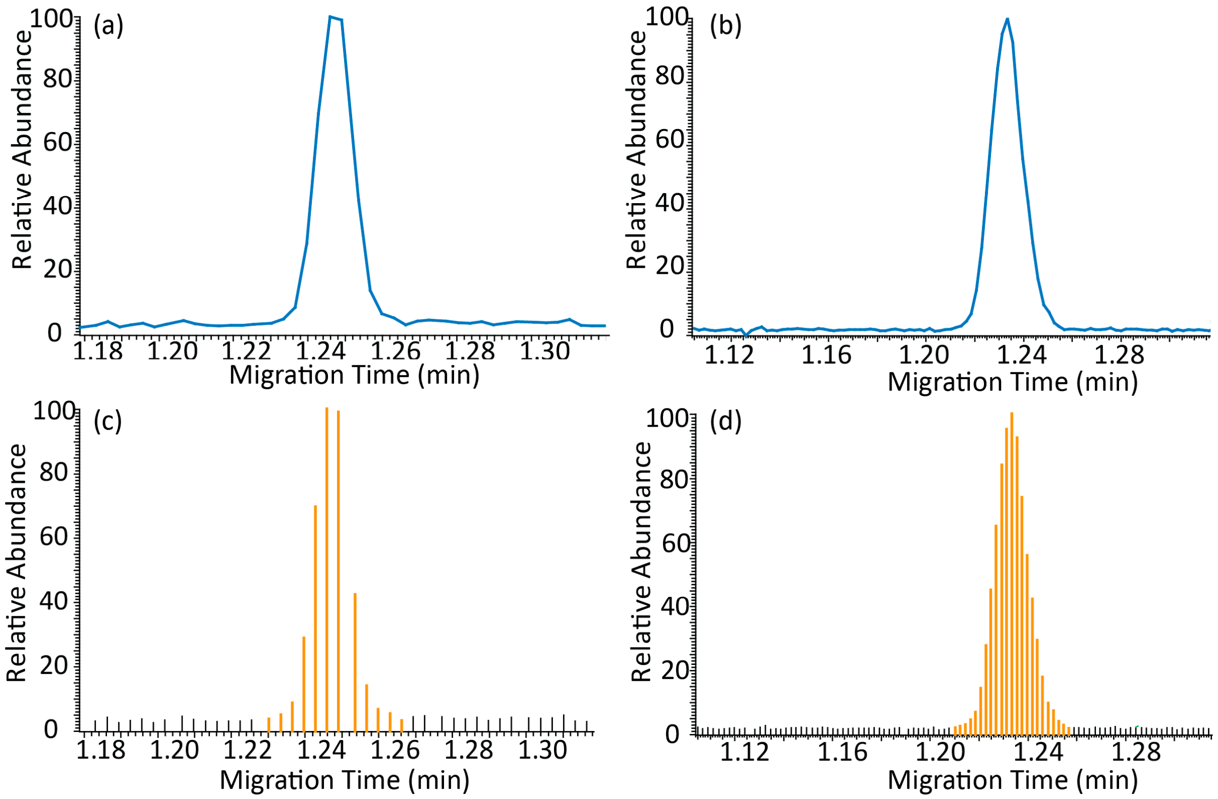

Figure S1. Comparison of µCE-HRMS peak shapes at different Orbitrap mass resolving power settings. Peak shape for alanine in a mixture of 20 analytes at (a) 35,000 resolving power (b) 17,500 resolving power. Individual MS scans acquired for alanine at (c) 35,000 resolving power (12 scans) and (d) 17,500 resolving power (24 scans).

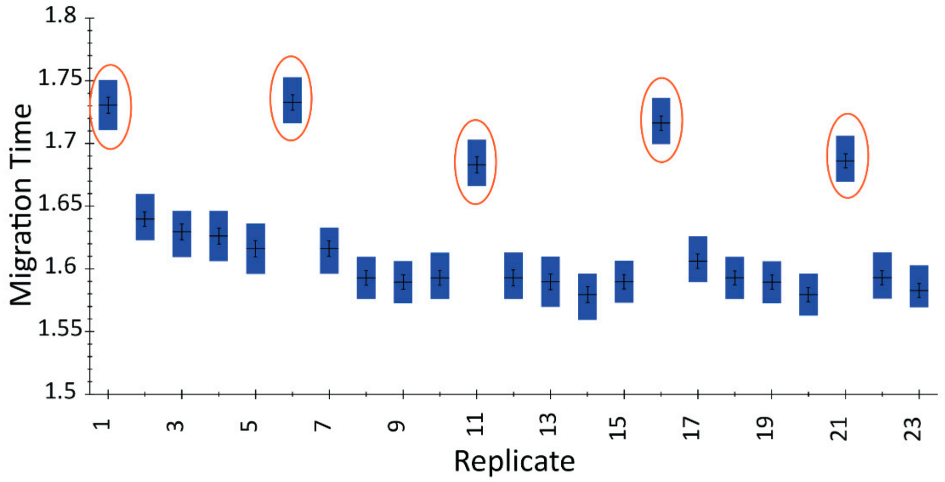

Figure S2. Analysis of migration time variation for metabolite analytes in TKO mouse serum. Migration time for ^{13}C phenylalanine across 23 replicates. The x-axis shows the replicate number and the y-axis represents the migration time window for the corresponding replicate. Replicates circled in orange represent the sample runs after background electrolyte (BGE) refreshes. Each replicate run lasted 3 min.

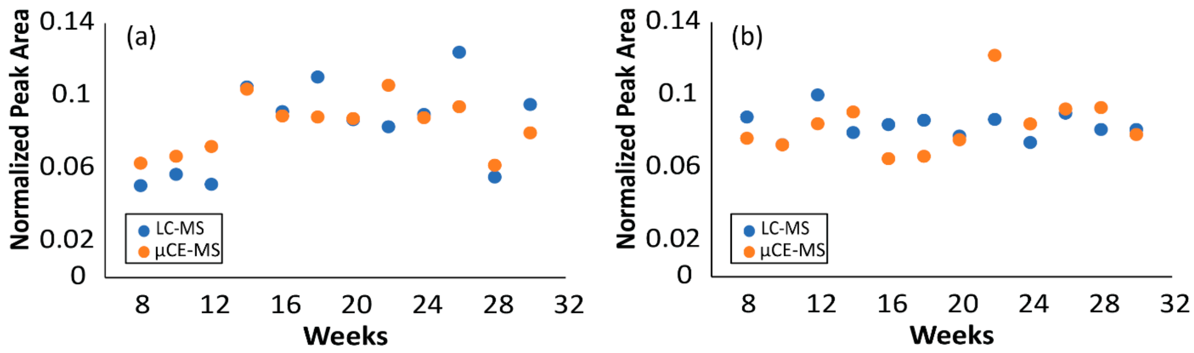

Figure S3. Comparison of time-resolved serum metabolomics data acquired with UHPLC-MS or µCE-HRMS platforms. Normalized peak abundance of (a) tryptophan and (b) ornithine in a TKO control mouse. The x-axis represents the age of mice in weeks. UHPLC-MS abundances are shown in blue and µCE-HRMS data in orange. For visualization purposes data were normalized to the sum of the peak areas for all time points.

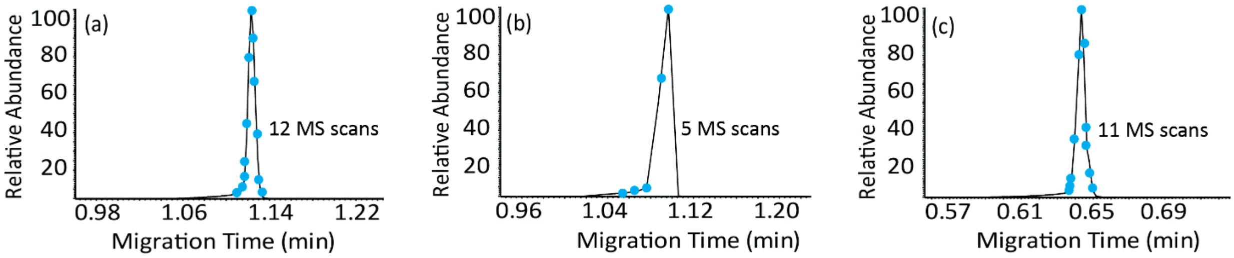

Figure S4. Comparison of µCE-HRMS peak shapes in different MS scan modes. Extracted ion electropherogram (XIE) of carnitine in (a) full scan mode (12 scans) , (b) parallel reaction monitoring (PRM) mode (5 scans) and (c) scheduled/multiplexed PRM mode (11 scans). MS scans per CE peak are shown as blue solid circles.

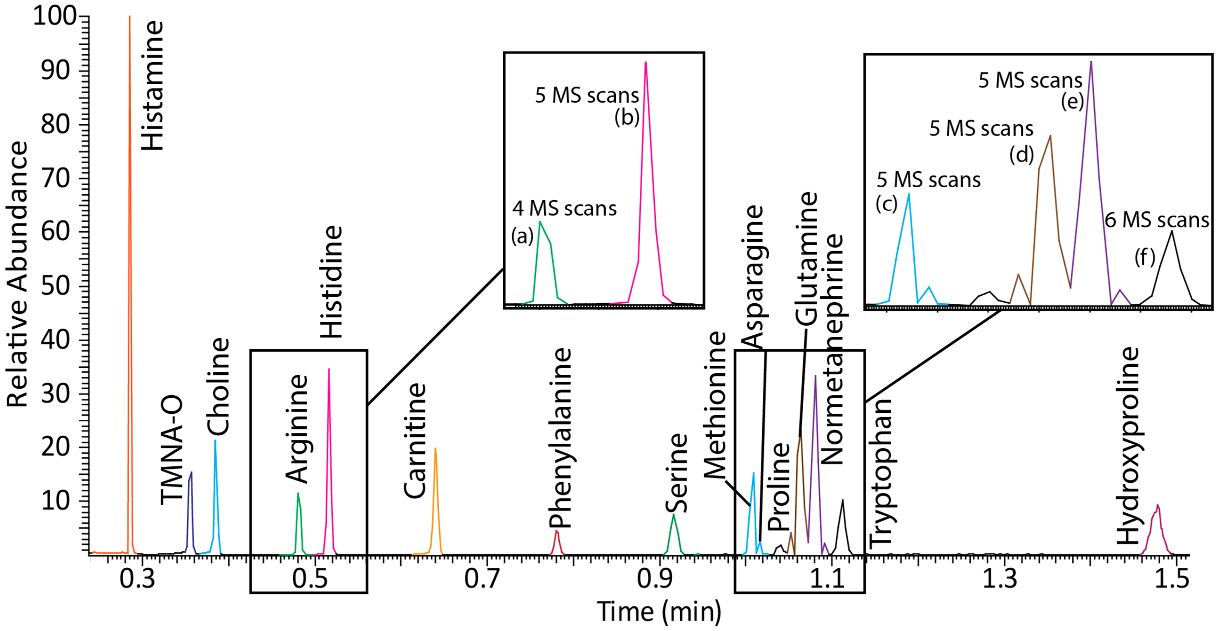

Figure S5. µCE-MS/MS separation of metabolites of interest. Extracted ion electropherogram (XIE) for a mixture of 15 metabolites in a scheduled/multiplexed PRM method. The x-axis represents the migration time in min and the y-axis the MS peak relative abundance. Individual MS scans acquired for (a) arginine (b) histidine (c) methionine (d) glutamine (e) normetanephrine and (f) tryptophan. Abbreviations : TMNA-O: trimethylamine-N-oxide.

Table S1: Metabolite standards and internal standards used for µCE-MS/MS analyses. Metabolite standards were purchased from Sigma Aldrich (Saint Louis, MO) and isotopically labeled internal standards were purchased from Cambridge Isotope Laboratories (Tewksbury, MA).

Chemical standard; CAS number	Internal standard used for data analysis; CAS number
5'-Hydroxy-L-tryptophan; 4350-09-8	^{13}C phenylalanine; 81201-86-7
Acetylcholine chloride; 60-31-1	^{13}C phenylalanine; 81201-86-7
L-Alanine; 56-41-7	^{13}C methionine D_3; 263568-73-6
2-Aminoisobutyric acid; 62-57-7	^{13}C phenylalanine; 81201-86-7
L-Arginine; 74-79-3	$^{13}C_6$ arginine; 201740-91-2
L-Asparagine; 70-47-3	$^{13}C_6$ arginine; 201740-91-2
Betaine; 107-43-7	^{13}C phenylalanine; 81201-86-7
L-Carnitine hydrochloride; 6645-46-1	^{13}C phenylalanine; 81201-86-7
Choline chloride; 67-48-1	$^{13}C_6$ arginine; 201740-91-2
N, N-Dimethylglycine; 1118-68-9	^{13}C phenylalanine; 81201-86-7
D-(+)-Glucosamine hydrochloride; 66-84-2	^{13}C phenylalanine; 81201-86-7
L-Glutamine; 56-85-9	$^{13}C_6$ arginine; 201740-91-2
Glycine; 56-40-6	^{13}C phenylalanine; 81201-86-7
L-Histidine; 71-00-1	$^{13}C_6$ arginine; 201740-91-2
Trans-4-hydroxy-L-proline; 51-35-4	^{13}C phenylalanine; 81201-86-7
L-Methionine; 63-68-3	^{13}C methionine D_3; 263568-73-6
DL-Normetanephrine hydrochloride; 1011-74-1	^{13}C phenylalanine; 81201-86-7
L-Phenylalanine; 63-91-2	^{13}C phenylalanine; 81201-86-7
L-Proline; 147-85-3	^{13}C phenylalanine; 81201-86-7
L-Serine; 56-45-1	$^{13}C_6$ arginine; 201740-91-2
Threonine; 72-19-5	^{13}C methionine D_3; 263568-73-6
Trimethylamine-N-oxide; 1184-78-7	^{13}C phenylalanine; 81201-86-7
L-Tryptophan; 73-22-3	^{13}C methionine D_3; 263568-73-6
L-Tyrosine; 60-18-4	^{13}C methionine D_3; 263568-73-6
L-Valine; 72-18-4	^{13}C methionine D_3; 263568-73-6
Nicotinamide; 98-92-0	^{13}C methionine D_3; 263568-73-6
L-Aspartic acid; 56-84-8	$^{13}C_6$ arginine; 201740-91-2
Nicotinic acid; 59-67-6	^{13}C phenylalanine; 81201-86-7
γ-Aminobutyric acid; 56-12-2	^{13}C phenylalanine; 81201-86-7
DL-2-Aminoadipic acid; 542-32-5	^{13}C phenylalanine; 81201-86-7
Cytidine; 65-46-3	$^{13}C_6$ arginine; 201740-91-2
L-Citrulline; 372-75-8	^{13}C methionine D_3; 263568-73-6
L-Kynurenine; 2922-83-0	^{13}C phenylalanine; 81201-86-7
L-Isoleucine; 73-32-5	^{13}C phenylalanine; 81201-86-7
L-Leucine; 61-90-5	^{13}C methionine D_3; 263568-73-6
L-Ornithine hydrochloride; 3184-13-2	$^{13}C_6$ arginine; 201740-91-2
L-Lysine; 56-87-1	^{13}C phenylalanine; 81201-86-7
Glutamic acid; 56-86-0	^{13}C methionine D_3; 263568-73-6
Adenosine; 58-61-7	^{13}C phenylalanine; 81201-86-7
Adenine; 73-24-5	^{13}C phenylalanine; 81201-86-7

Table S2: Multiplexed/scheduled µCE-MS/MS method. MS sampling frequency across µCE peaks and %RSD of peak areas in a scheduled/multiplexed parallel reaction monitoring (PRM) method for 12 metabolites are shown. Most relevant MS parameters: AGC target value 1E5 and maximum ion injection time of 20 ms. Spectral multiplexing (MSX) count of 6.

**Metabolite**	**Peak Width (seconds)**	**Number of MS scans across peak**	**Peak Area %RSD without internal standard correction (*n*=4)**
Arginine	1.2	7	11
Histidine	1.2	7	14
Carnitine	1.8	10	10
Normetanephrine	1.8	12	7
Serine	1.8	12	9
Methionine	1.8	9	7
Proline	1.8	10	11
Phenylalanine	1.8	10	12
Tryptophan	1.8	10	11
Glutamine	2.1	10	8
Trans-hydroxyproline	3.0	15	8

Table S3. Migration times and relative standard deviation (RSD) for peak areas of target analytes in TKO serum.

**Metabolite Name**	**Migration time (min)**	**%RSD for peak area without internal standard correction (*n*=23)**	**%RSD for peak area of metabolite/ ^{13}C phenylalanine (*n*=23)**	**%RSD for peak area of metabolite / $^{13}C_6$ arginine (*n*=23)**	**%RSD for peak area of / ^{13}C methionine D_3**
Alanine	1.2	17.24	6.21	10.46	6.07
Aminoisobutyric acid	1.3	21.92	4.91	10.93	5.55
arginine	1.0	20.86	12.18	2.85	12.25
asparagine	1.6	25.92	15.24	13.37	15.47
Betaine	1.9	17.14	6.75	10.42	7.67
Carnitine	1.2	15.12	11.45	12.62	12.67
Choline	0.9	27.72	23.28	19.39	23.55
Dimethylglycine	1.8	18.22	4.56	10.71	5.55
Glutamine	1.7	20.47	11.19	10.95	11.26
Glycine	1.2	20.38	8.25	10.44	8.79
Histidine	1.1	22.35	10.39	9.59	11.08
Trans-hydroxyproline	2.0	21.22	8.50	8.64	8.73
Methionine	1.5	20.61	4.50	11.99	1.11
Phenylalanine	1.6	20.65	0.93	11.77	4.17
Proline	1.6	17.94	4.37	12.64	4.44
Serine	1.5	27.30	14.47	13.09	14.56
Threonine	1.5	23.20	8.99	9.93	8.34
Tryptophan	1.6	24.01	7.84	10.49	7.27
Tyrosine	1.8	25.05	10.75	11.65	10.09
Valine	1.4	20.60	4.77	10.41	4.66
Nicotiniamide	1.5	21.28	5.33	10.10	4.48
Aspartic acid	1.8	26.43	14.03	12.96	14.57
Aminoadipic acid	1.7	21.31	8.27	11.11	8.42
Cytidine	1.5	27.08	15.16	14.47	15.21
Ornithine	1.0	24.58	14.82	12.45	14.33
Citrulline	1.7	24.19	11.20	12.21	11.13
Isoleucine	1.4	23.18	7.01	10.26	7.33
Leucine	1.4	22.26	6.87	9.74	6.21

Lysine	1.0	22.24	13.79	14.34	13.91
Glutamic acid	1.7	23.87	11.81	12.29	11.74
^{13}C phenylalanine	1.6	20.99	--	--	--
$^{13}C_6$ arginine	1.0	19.83	--	--	--
^{13}C methionine D_3	1.5	20.92	--	--	--

Table S4. TKO mouse cohort information. The mouse ID, mouse age in weeks and corresponding %lifetime values are given for each mouse used in the analysis.

Mouse ID	Phenotype	CE-MS sample ID	Age (weeks)	% Lifetime
8465	TKO	T13_1	8	33.0%
8465	TKO	T13_2	10	42.0%
8465	TKO	T13_3	12	50.0%
8465	TKO	T13_4	14	58.0%
8465	TKO	T13_5	16	67.0%
8465	TKO	T13_6	18	75.0%
8465	TKO	T13_7	20	83.0%
8465	TKO	T13_8	22	92.0%
8465	TKO	T13_9	24	100.0%
8256	TKO	T11_1	8	22.0%
8256	TKO	T11_2	10	28.0%
8256	TKO	T11_3	12	33.0%
8256	TKO	T11_4	14	39.0%
8256	TKO	T11_5	16	44.0%
8256	TKO	T11_6	18	50.0%
8256	TKO	T11_7	20	56.0%
8256	TKO	T11_8	22	61.0%
8256	TKO	T11_10	26	72.0%
8256	TKO	T11_11	28	78.0%
8256	TKO	T11_12	30	83.0%
8256	TKO	T11_13	32	89.0%
8256	TKO	T11_14	34	94.0%
8256	TKO	T11_15	36	100.0%
8472	TKO	T15_1	8	27.0%
8472	TKO	T15_3	12	40.0%
8472	TKO	T15_4	14	47.0%
8472	TKO	T15_5	16	53.0%
8472	TKO	T15_6	18	60.0%
8472	TKO	T15_7	20	67.0%
8472	TKO	T15_8	22	73.0%
8472	TKO	T15_9	24	80.0%
8472	TKO	T15_10	26	87.0%
8472	TKO	T15_11	28	93.0%
8103	Control	C8_1	8	24.0%
8103	Control	C8_2	10	29.0%
8103	Control	C8_3	12	35.0%
8103	Control	C8_4	14	41.0%
8103	Control	C8_5	16	47.0%
8103	Control	C8_6	18	53.0%

8103	Control	C8_7	20	59.0%
8103	Control	C8_8	22	65.0%
8103	Control	C8_9	24	71.0%
8103	Control	C8_10	26	76.0%
8103	Control	C8_11	28	82.0%
8103	Control	C8_12	30	88.0%
8103	Control	C8_13	32	94.0%
8103	Control	C8_14	34	100.0%
8284	Control	C10_1	8	27.0%
8284	Control	C10_2	10	33.0%
8284	Control	C10_3	12	40.0%
8284	Control	C10_4	14	47.0%
8284	Control	C10_5	16	53.0%
8284	Control	C10_6	18	60.0%
8284	Control	C10_7	20	67.0%
8284	Control	C10_8	22	73.0%
8284	Control	C10_9	24	80.0%
8284	Control	C10_10	26	87.0%
8284	Control	C10_11	28	93.0%
8284	Control	C10_12	30	100.0%
8512	Control	C13_1	8	25.0%
8512	Control	C13_2	10	31.0%
8512	Control	C13_3	12	38.0%
8512	Control	C13_4	14	44.0%
8512	Control	C13_5	16	50.0%
8512	Control	C13_6	18	56.0%
8512	Control	C13_7	20	63.0%
8512	Control	C13_8	22	69.0%
8512	Control	C13_9	24	75.0%
8512	Control	C13_10	26	81.0%
8512	Control	C13_11	28	88.0%
8512	Control	C13_12	30	94.0%
8512	Control	C13_13	32	100.0%
